# Spatial transcriptomic data denoising and domain identification by a community strength-augmented graph autoencoder

**DOI:** 10.1093/bib/bbaf540

**Published:** 2025-10-10

**Authors:** Ke Huang, Wenqian Tu, Lihua Zhang

**Affiliations:** School of Artificial Intelligence, School of Computer Science, Wuhan University, No. 299 Bayi Road, Wuhan 430072, China; School of Artificial Intelligence, School of Computer Science, Wuhan University, No. 299 Bayi Road, Wuhan 430072, China; School of Artificial Intelligence, School of Computer Science, Wuhan University, No. 299 Bayi Road, Wuhan 430072, China

**Keywords:** spatial transcriptomics, domain identification, graph contrastive learning

## Abstract

The rapid development of spatial sequencing technologies has generated large amounts of spatial transcriptomic data, which provide an opportunity to explore complex tissue structures and functional domains. However, such data often suffer from high noise and sparsity, which bring a big challenge for deciphering spatial domains and further understanding the structural and functional organization of biological tissues. In this study, we propose a novel method named Community Strength-Augmented (CSA) that incorporates community strength-augmented graph autoencoder by considering spatially heterogenous structures. Moreover, attention mechanism is designed in CSA to take full advantage of both spatial transcriptomic data and corresponding histology image information. We applied CSA to several spatial transcriptomic datasets derived from various platforms. Compared with the state-of-the-art methods, CSA exhibits superiority in revealing spatially functional domains. Moreover, CSA is able to denoise the data, enabling the identification of biologically meaningful marker genes.

## Introduction

Recently, remarkable progress achieved in spatially resolved transcriptomics (SRTs) has advanced gene expression profiling with spatial information in tissues [1–3]. Spatial information of cells or spots in a tissue is crucial for researchers to understand the internal relationships between cells and the surrounding environment. Current spatial transcriptomics technologies can be broadly categorized into two groups: *in situ* hybridization (ISH)-based methods and spatial barcoding-based methods [4]. These technologies differ in gene throughput and resolution. ISH-based technologies, such as seqFISH [5, 6] and multiplexed error-robust fluorescence ISH (MERFISH) [7], detect target transcripts at subcellular resolution, while spatial barcoding-based methods can capture total transcriptome at different spatial point resolutions. For example, widely used 10× Visium [8] measures spots containing around 10 cells with ~55 μm diameter. Slide-seqV2 [9] accomplishes the same task with a diameter of ~10 μm. The recently emerged Seq-Scope [10], utilizing the Illumina platform, reduces the diameter further to 0.5–0.8 μm. Nevertheless, the large-scale, high-resolution data generated by these technologies are typically accompanied by elevated noise.

Identifying spatial domains (i.e. regions with the same spatial expression pattern) is a crucial task in SRT analysis study. Existing methods are broadly classified as nondeep-learning or deep-learning approaches. Nondeep-learning-based methods (e.g. Giotto [11], BayesSpace [12]) employ probabilistic graphical models to precisely localize spatial domains with similar gene expression patterns. UTAG [13] aggregates molecular features via message passing for cell-type assignment, whereas SC-MEB [14] leverages empirical Bayes and hidden Markov random fields for spatial clustering. However, probabilistic graphical models have difficulties in efficiently handling high-dimensional data and capturing complex hierarchical relationships. To address these challenges, some deep-learning-based models have been proposed. STAGATE [15] utilizes a graph attention network to adaptively learn the similarity of neighboring spots. SpaGCN [16] and SpaceFlow [17] make use of graph convolution network to integrate gene expression and spatial location information. GraphST [18] uses self-supervised graph contrastive learning (GraphCL) to perform spatially informed clustering. CytoCommunity [19] implements a MinCut-based graph neural network model to identify tissue domains, and SiGra [20] employs an image-augmented graph-transformer to denoise single-cell spatial transcriptomic profiles. Extending SiGra, xSiGra [21] incorporates an interpretable Grad-CAM module to identify key genes and cells underlying each spatial cell type. Most of these methods do not take advantage of histology image, which may provide complementary details for gene expression data, especially for spatial transcriptomics data of cancer. Moreover, the commonly used GraphCL in such methods treats each spot equally when generate corrupted graph without considering the inherent heterogeneous characteristic, which may lead to limited performance in analysing spatial transcriptomics data.

To fully consider the inherently complex structures among spots in SRT data, we propose a Community Strength-Augmented (CSA) graph autoencoder method CSA. CSA adopts GraphCL to extract spatially aware embedding of SRT data with augmented graphs being generated by considering community strength. We applied CSA to several real datasets including spatial transcriptomics data coming from 10× Visium, ST, Slide-SeqV2, and MERFISH platforms. Comprehensive benchmark analyses with other methods demonstrate the superiority of CSA in identifying spatial domains and denoising spatial transcriptomics data.

## Materials and methods

**Figure 1:**
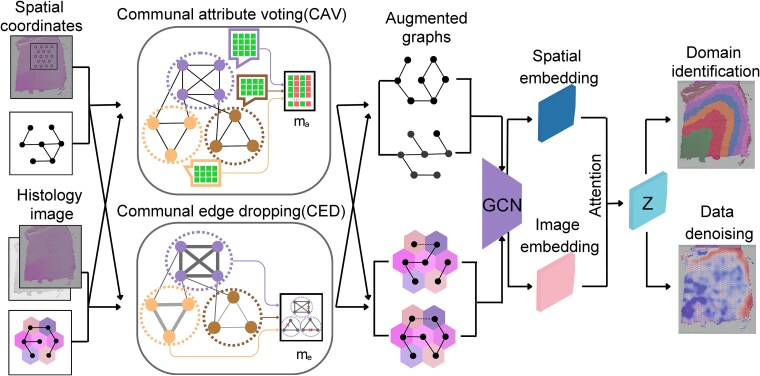
Workflow of CSA. CSA builds spatial graph and histology graph based on the spatial coordinates and histology image (if available), respectively. We treat the processed gene expression as nodes’ features. Then we generated two augmented views for each graph by masking nodes and edges based on community strength. Then latent embeddings are learned by graph contrastive learning by taking advantages of both spatial coordinates or histology image and gene expression information. An attention layer is used to obtain the final embedding and a GCN decoder is employed to reconstruct gene expression values. The final embedding is used for downstream analysis, such as spatial domain identification.

CSA takes spatial coordinates, gene expression, and optional histology image as input (Fig. 1). It first constructs graphs based on spatial coordinates and histology image, respectively. Each node corresponds to a spot, with gene expression values as features. Next, CSA generates two augmented views based on community strength on each graph (Methods). A Graph Convolutional Network (GCN) [22] is used as an encoder to learn the low-dimensional embedding of each view. These embeddings are then adaptively integrated by an attention layer to obtain the final latent embedding, which is used to identify spatial domains by algorithms such as Louvain [23], Leiden [24], and mclust [25]. Simultaneously, a GCN decoder is used to reconstruct the expression matrix.

### Datasets and data preprocessing

In this study, we applied CSA to four spatial datasets from different platforms, including 10× Visium, ST, MERFISH, and Slide-seqV2. The DLPFC dataset consists of 12 human sections obtained from 10× Visium platform [26]. PDAC is a human primary pancreatic cancer dataset generated by the ST technology dataset [27]. Healthy mouse hypothalamic preoptic dataset was generated using the multiplexed error-robust fluorescence ISH (MERFISH) technology to measure the expression quantity of 155 genes [28]. The MOB dataset is cellular-level data, which is generated by Slide-seqV2 platform [29]. The gene expression data is normalized and log-transformed using Scanpy package. Top 3000 highly variable genes are selected. Image features are extracted by utilizing the image container function in Squidpy package.

### Building graphs

We constructs the spatial graph *G_s_* using alpha-complex method [30] based on spatial coordinates. Specially, we calculates a unique site (Voronoi cell $\boldsymbol{V}(r)$ for each spot $r$ using the alpha complex method [31] as follows:


$$ \boldsymbol{V}(r)=\left\{x\in{\mathbb{R}}^2|\ \left\Vert x-r\right\Vert \le \left\Vert x-{r}^{\prime}\right\Vert, \forall{r}^{\prime}\in S\right\}\!. $$

where $V(r)$ is a spot set composed of any point $x$ which is closer to $r$ than to any other point ${r}^{\prime }$. $S$ is the set of coordinates for all spots and $\left\Vert \bullet \right\Vert$ is the cosine distance between two spots. Next, we identify edges *E* of *G_s_* by connecting spots *i* and *j* as follows:


$$ E=\left\{\left({v}_i,{v}_j\right)|\ \bigcap_{k\in \left\{{v}_i,{v}_j\right\}}\left(V(k)\cap C\left(k,{\sigma} \right)\right)\right\}, $$

where $C\left(k,{\sigma} \right)$ is a set of neighborhood spots whose center point coordinate is $k$ with a radius $\sigma$. The radius $\sigma$ is estimated from the average distance of k-nearest-neighbors (KNN) [32] of each spot.

When histological image is available especially for the spatial transcriptomics data of cancer, we construct histology graph ${G}_h$ based on the image features. Specially, we computed the KNN of the image feature vector for each spot by calculating Euclidean distance on image features. Then we combine the spatial graph and histology graph by ${G}_w={G}_s\ast{w}_s+{G}_h\ast{w}_h+I$, where $I$ is the identity matrix, ${w}_s$ and ${w}_h$ are the weights of spatial graph and histological graph, respectively. And the default values are set to 0.5 and 0.5.

### Graph augmentations based on community strength

As the annotation information of the spatial transcriptomics data is usually unavailable, we adopt GraphCL method to model the spatial transcriptomics data. While the commonly used GraphCL builds augmented graphs by randomly mask edges or nodes. It treats each spot from tissues equally without considering the heterogeneously spatial structures within a tissue. Therefore, we incorporated community-enhanced strategies {Communal Attribute Voting (CAV) [33] and Edge Dropping (CED)} on edges and nodes to generate the augmented graphs. A community of a graph, proposed by Newman and Girvan [34], can be defined as a subset of nodes that are densely connected to each other and loosely connected to the nodes of other communities in the same graph. The community strength is described as follow:


$$ {S}_c=\frac{\mid{\varepsilon}_c\mid }{\mid \varepsilon \mid }-\frac{\left[(\sum_{v\in c}d(v)\right]{}^2}{4{\left|\varepsilon \right|}^2}, $$

where $\varepsilon$ is the number of edges of the entire graph and ${\varepsilon}_c$ is the number of edges of the community *c*. The initial communities are detected by Leiden algorithm on the normalized gene expression profile with default resolution value equaling 1.

Each spot will participate in the voting for each selected gene on whether to remove or retain. During CAV, a community penalty ${p}_a$ is calculated as follows:


$$ {p}_a=\overline{n_a}\left(\mathit{\log}\left( abs(X) IS\right)\right), $$

where $\overline{n_a} = \left({x}_{max}-x\right)/\left({x}_{max}-{x}_{mean}\right)$ is a normalization operation, $I\in{\left\{0,1\right\}}^{\mathrm{n}\times \mid C\mid }$ is an indicator matrix indicates each node belongs to which community. Then we compute the corruption level [35] for each single gene. Specially, let ${m}_a^{(i)}\left(i=1,2\right)$ represent the masked probability for the gene expression matrix, which is independently sampled from Bernoulli distribution with the hyper-parameter ${\lambda}_a^{(i)}\left(i=1,2\right)$ as follows:


$$ {m}_a^{(i)}\sim Bernoulli\left(1-{p}_a{\lambda}_a^{(i)}\right),i=1,2 $$

With the masked probability of expression matrix, we can obtain the corrupted matrix by Hadamard product operation:


$$ {\overset{\sim }{X}}^{(i)}={m}_a^{(i)}\odot X,i=1,2 $$

Next, we apply the edge dropping graph augmentation which is evolved from DropEdge [36] to get the edge corruption of the augmented graphs. Intra-community edges are given higher significance than inter-community edges. The assumption of CED is that interactions between communities play a crucial role in communication within different tissue areas. Edges within strong communities are considered more important than those within weak communities. Specially, the edge weight of $e=\left({v}_i,{v}_j\right)$ is computed by:


$$ {p}_e=\left\{\begin{array}{@{}c}\overset{\sim }{n_e}\left({I}_iS\right),{\left(I\bullet{I}^T\odot A\right)}_{i,j}==1\ \\{}-\overset{\sim }{n_e}\left({I}_iS+{I}_jS\right), otherwise\end{array}\right. $$

where $\overset{\sim }{n_e}$ is a normalization function similar to $\overset{\sim }{n_a}$ and $I\in{\left\{0,1\right\}}^{\mathrm{n}\times \mid C\mid }$. Let ${m}_e^{(i)}\left(i=1,2\right)$ represent the masked probability for the edge, which is independently sampled from Bernoulli distribution with the hyper-parameter ${\lambda}_e^{(i)}\left(i=1,2\right)$ as follows:


$$ {m}_e^{(i)}\sim Bernoulli\left(1-{p}_e{\lambda}_e^{(i)}\right),i=1,2 $$

The corrupted adjacency matrix ${\overset{\sim }{A}}^{(i)},\left(i=1,2\right)$ for the augmented graphs can be obtained by the follows:


$$ {\overset{\sim }{A}}^{(i)}=\left[{\left({m}_e^{(i)}\right)}_{j,k}{A}_{j,k}^{(i)}\right]\left(i=1,2;j,k\in \left|V\right|\right). $$

### Graph contrastive learning paradigm

After two augmented graphs are obtained, we adopt a two-layer GCN encoder to extract low-dimensional embeddings ${Z}^{(1)}$ and ${Z}^{(2)}$ on the augmented graphs using community-strength-enhanced InfoNCE [37]. The contrastive loss function is defined as follows:


$$ L={\mathbb{E}}_{\left({Z}^{(1)},{Z}^{(2)}\right)}\left(-\frac{1}{n}\sum_{i=1}^n\mathit{\log}\frac{\exp \left({\tilde{s}}_{ii}^{\left(1,2\right)}\right)}{\sum_{j=1,j\ne i}^n{\tilde{s}}_{ij}^{\left(1,1\right)}+\sum_{j=1}^n{\tilde{s}}_{ij}^{\left(1,2\right)}}\right), $$

where ${s}_{ij}^{\left(1,2\right)}= sim\left({Z}_{i:}^1,{Z}_{j:}^2\right)/\tau$. $sim\left({z}_i,{z}_j\right)=\frac{z_i^T{z}_j}{\parallel{z}_i\parallel \parallel{z}_j\parallel }$. $\gamma (k)$ was a dynamic balancing coefficient that could gradually increase during training $\gamma \left(k;{k}_0,{\gamma}_{max}\right)=\mathit{\min}\left\{\mathit{\max}\left\{0,k-{k}_0\right\},{\gamma}_{max}\right\}$.

After obtaining the latent embeddings for spatial, histology graph from the GCN encoder, CSA effectively integrates them with an adaptive attention layer [38] to get the final latent embedding *Z*. The attention layer can be described as follow:


$$ {Z}_f=\left[{Z}_s\mid |\, {Z}_h\right], $$

$$ \alpha = Softmax\left({W}_2\left(\mathit{\tanh}\left({W}_1{Z}_f+{b}_1\right)\right)+{b}_2\right), $$

$$ Z=\alpha \ast{Z}_f, $$

where ${Z}_s,{Z}_h$ are latent embeddings for spatial and histology graphs.$\Vert$ represents the operation of tensor stacking, ${W}_1$ and ${W}_2$ are linear layers with bias ${b}_1$ and ${b}_2$_,_  $\mathit{\tanh}\left(\right)$ is an activation function. $Softmax\left(\right)$ converts values into contribution scores by squashing the input values into the range (0, 1) and normalizes them so that they sum up to 1.

The embedding *Z* is fed into GCN decoder on fused graph ${G}_w$to denoise the gene expression profile. The corresponding loss function is:


$$ {\mathcal{L}}_{recon}=\left\Vert f(Z)-X\right\Vert, $$

where $X$ is the normalized gene expression matrix.

The total loss function in our study can be formulated as:


$$ {L}_{total}={\lambda}_{topo}{L}_{topo}+{\lambda}_{HE}{L}_{HE}+{\lambda}_{recon}{L}_{recon}, $$

where ${L}_{topo}$ is topology contrastive loss, ${L}_{HE}$ is histology contrastive loss and ${L}_{recon}$ is the reconstruction loss. In this study, we adopted the following default parameters: ${\lambda}_{topo}=0.1,{\lambda}_{HE}=0.1,{\lambda}_{recon}=1.0$. When H&E image is not available, we set ${\lambda}_{HE}$ to 0. We performed the experiments on Intel(R) Xeon(R) E5-2640 v4 CPU with 20 cores and Nvidia Tesla V100 GPU. Specifically, it costs less than 1 min per 100 epochs and needs ~11953.36 MB memory on the largest data with 20 139 spots (Supplemental Table S1).

## Results

### CSA shows accuracy and robustness in spatial domain detection of human dorsolateral prefrontal cortex

**Figure 2:**
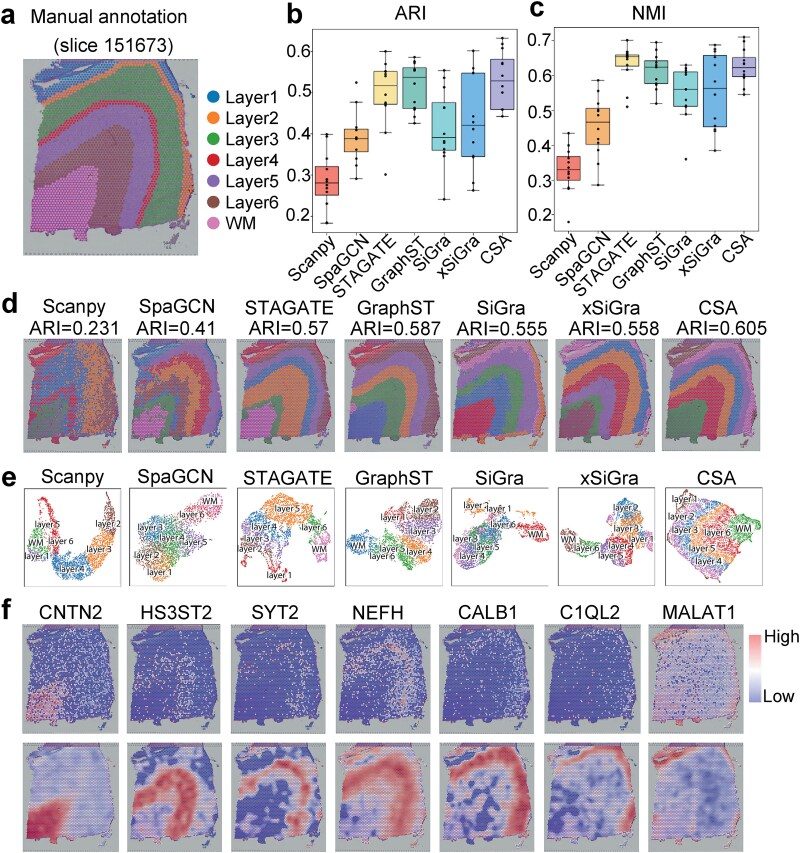
Benchmarking CSA against other methods on 10× Visium DLPFC dataset. (a) Manual annotation of section 151673 including six cortical layers and the WM layer. (b) ARI of the domains identified by mclust on the embeddings of Scanpy, SpaGCN, STAGATE, GraphST, SiGra, xSiGra, and CSA on all 12 sections. In the boxplot, the center line, box upper marker line, lower marker line and whiskers denote the median, upper and lower quartiles, and 1.5× interquartile range, respectively. (c) NMI of the domains identified by mclust on the embeddings of Scanpy, SpaGCN, STAGATE, GraphST, SiGra, xSiGra, and CSA on all 12 sections. (d) Spatial visualization of the domain identification results on section 151673 by the seven methods. The spots are colored by the annotation label. (e) UMAP visualization of the graph representation embedding vectors obtained by the seven methods. The spots are colored by the annotation label. (f) Spatial visualization of raw spatial gene expression patterns (top) and denoised gene expression patterns by CSA (bottom) of CNTN2, HS3ST2, SYT2, NEFH, CALB1, C1QL2, and MALAT1, corresponding to WM layer and layers 1 to 6 respectively.

We first applied CSA to the 10× Visium human dorsolateral prefrontal cortex (DLPFC), which comprises 12 sections and each section contains 5–7 cortical layers including white matter (WM) (Fig. 2a). Using annotated labels as ground truth, we compared CSA with six other methods including Scanpy [39], SpaGCN, STAGATE, GraphST, SiGra, and xSiGra. We evaluated these methods using the Normalized Mutual Information (NMI) [40] and the Adjusted Rand Index (ARI) [41] (Fig. 2b and c) across all the 12 sections. Among all the methods, CSA exhibits top-tier performance, consistently ranking within the top two. Moreover, we substituted the node and edge perturbation strategies in CSA with those from GraphCL, designating them as GraphCL-F, and GraphCL-E, respectively. CSA had higher ARI and NMI scores than GraphCL-F and GraphCL-E (Supplementary Fig. S1 and S2). There were no obvious patterns detected on the histology image (Supplementary Fig. S3). Therefore, integrating histology image did not improve the performance of CSA and we did not leverage histology image on this data (Supplementary Fig. S4).

Next, we provided a detailed analysis of the DLPFC section 151673 (Fig. 2d). CSA successfully delineated the expected seven cortical layers, including the WM layer with clear boundary demarcations. We applied mclust on the embedding of CSA and found that CSA achieved the higher ARI value than other methods. STAGATE generated irregular boundaries between WM and layer 6; GraphST failed to detect the anatomically narrow layer 2 and xSiGra failed in layer 1 identification. In contrast, CSA achieved complete detection of both layer 1 and layer 2, with well-defined borders. Nonspatial Scanpy partially matched the expected layers but exhibited frequent outliers and discontinuous boundaries.

Additionally, we visualized the low-dimensional embedding of section 151673 of CSA and other methods using Uniform Manifold Approximation and Projection (UMAP) technique [42]. Both CSA and STAGATE demonstrated well-ordered and continuous cortical layers, while CSA better captured the inherent hierarchical structure of the section (Fig. 2e). By comparison, the UMAP plots from SpaGCN and Scanpy showed poorly differentiated clusters of neighboring layers. Although SiGra and xSiGra achieved comparable accuracy in spatial domain identification, their low-dimensional embeddings exhibited discontinuous patterns in UMAP. CSA also demonstrated robust data denoising capabilities on the DLPFC data. We depicted the expression patterns of representative marker genes for each layer. Consistent with the annotated layers, CSA significantly denoised the marker genes’ expression compared to the raw expression values (Fig. 2f). For example, the denoised expression of MALAT1 and C1QL2 are markedly enriched in Layer1 and Layer2 respectively, which provided the basis for identifying these two narrow regions.

### CSA accurately delineates the cancerous and noncancerous regions of human primary pancreatic cancer

**Figure 3:**
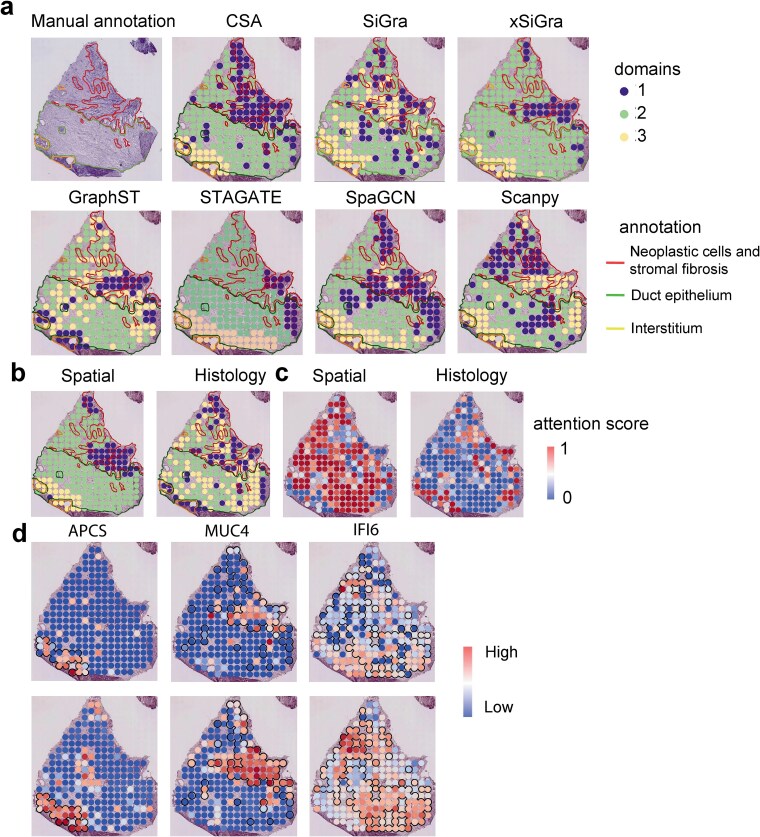
The performance of CSA on the human primary pancreatic cancer tissue data. (a) Spatial visualization of manually annotated regions, and domains identified by mclust on the embeddings of CSA, SiGra, xSiGra, SCANPT, GraphST, SpaGCN, and STAGATE. Red lines mark neoplastic cells and stromal fibrosis, green lines mark ductal epithelium, and yellow lines mark the interstitium. (b) Ablation experiments on PDAC data. Spatial visualization of domains identified by CSA with only spatial coordinates (left) and histology image (right). (c) Spatial visualization of attention scores of spatial and histological information in CSA. (d) Spatial visualization of marker genes MMP11, SPINK1, and AEBP1, which marked the neoplastic cells and stromal fibrosis region, the interstitium region and the duct epithelium respectively. To highlight the relationship between the high-expression regions of these marker genes and the spatial domains identified by CSA, the domains were manually outlined.

Next, we evaluated CSA on a human primary pancreatic cancer dataset generated using ST technology. We benchmarked CSA against seven other methods, including Scanpy, SpaGCN, STAGATE, GraphST, SiGra, and xSiGra. This dataset comprises 16 448 genes across 224 spots and pathologist-annotated labels are available [27]. The spots include neoplastic cells and stromal fibrosis, duct epithelium and interstitium. We utilized ImageContainer function of Squidpy [43] to extract the features of the pathological image and integrated these features with spatial information. CSA outperformed benchmark methods in distinguishing cancerous versus noncancerous regions (Fig. 3a). It also performed better in identifying interstitium region. In comparison, both Scanpy and STAGATE failed to consistently identify the cancerous areas. SpaGCN inaccurately categorized certain noncancerous areas as cancerous. Both GraphST and xSiGra achieved similar identification results, detecting only partial cancerous regions.

We conducted ablation study for CSA to check the advantage of integrating histological data. Models trained without spatial coordinates or histology images failed to detect correct domains (Fig. 3b). Adaptive attention layer was used in CSA, which could learn the relative importance of spatial and histological information. Therefore, we visualized the attention scores to explore the contribution of the spatial and histological information. We found that histological attention scores were highly enriched in the cancerous regions, which the spatial attention scores were highly enriched in the epithelium region (Fig. 3c). GraphCL-F and GraphCL-E failed to detect the neoplastic cells and stromal fibrosis (Supplementary Fig. S5). CSA also demonstrated remarkable data denoising ability in PDAC data. The cancer-associated marker gene MUC4 showed further enrichment in CSA-denoised data, closely matching annotated cancerous regions (Fig. 3d).

### CSA performs well in identifying tissue structures of spatial transcriptomics data of MERFISH platform

**Figure 4:**
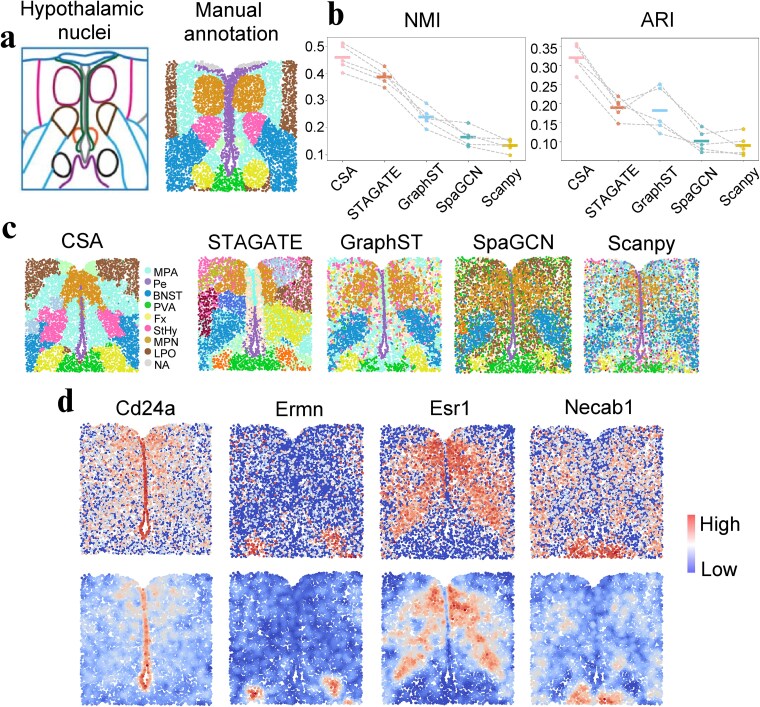
The performance of CSA on the spatial transcriptomics data of mouse hypothalamic preoptic region. (a) the organization of hypothalamic nuclei (left) and manually assigned domains according to the nuclei region (right) for the slice Bregma −0.14. (b) Comparation of the clustering performance of Louvain on the embeddings of CSA, STAGATE, GraphST, SpaGCN, and Scanpy using NMI (left) and ARI (right). Scores for the same slice across different methods are connected with gray dashed lines for clearer comparison. (c) Spatial visualization of the domains identified by CSA, STATAGE, GraphST, SpaGCN, and Scanpy. The domains include periventricular hypothalamic nucleus (Pe), the BNST region, the StHy region, MPA, MPN, ParaVentricular thalamic nucleus (PVA), Fx, MPA, and lateral preoptic area (LPO). (d) Comparison of the raw expression levels (top) and the denoised gene expression levels (bottom) of Cd24a, Ermn, Esr1, and Necab1.

We applied CSA to the spatial transcriptomics data of mouse hypothalamic preoptic region generated by MERFISH technology and compared it against GraphST, STAGATE, SpaGCN, and Scanpy. We analyzed five samples from different brain regions including Bregma −0.14, Bregma −0.04, Bregma +0.06, Bregma +0.16, and Bregma +0.26. These samples spanned 17 hypothalamic nuclei regions in the original study [44]. We manually annotated spots based on hypothalamic nuclei regions and used these annotations as ground truth (Fig. 4a). We evaluated clustering performance by applying the Louvain algorithm to embeddings from CSA and four competing methods (STAGATE, GraphST, SpaGCN, and Scanpy), calculating NMI and adjusted ARI scores. CSA achieved significantly higher NMI and ARI scores than all benchmark methods (Fig. 4b). Moreover, CSA had higher NMI and ARI scores than GraphCL-E and GraphCL-F (Supplementary Fig. S6).

We focused on the Bregma −0.14 slice as a representative example. CSA generated anatomically consistent spatial domains, whereas other methods showed fragmentation (Fig. 4c, Supplementary Fig. S7). Specifically, CSA accurately identified the bed nucleus stria terminalis (BNST) and striohypothalamic nucleus (StHy) regions, whereas STAGATE classified them into separate categories. Although GraphST produced partially consistent domains, it missed the StHy region entirely. Although GraphST produced partially consistent domains, it missed the StHy region entirely. SpaGCN and Scanpy provided a coarse identification of the structures. Moreover, CSA accurately recovered the gene expression patterns of Cd24a, Ermn, Esr1, and Necab1, which corresponded to domain periventricular hypothalamic nucleus (Pe), fornix (Fx), medial preoptic nucleus (MPN), and paraventricular thalamic nucleus (PVA) respectively (Fig. 4d, Supplementary Fig. S8).

### CSA reveals the laminar organization of the mouse olfactory bulb

**Figure 5:**
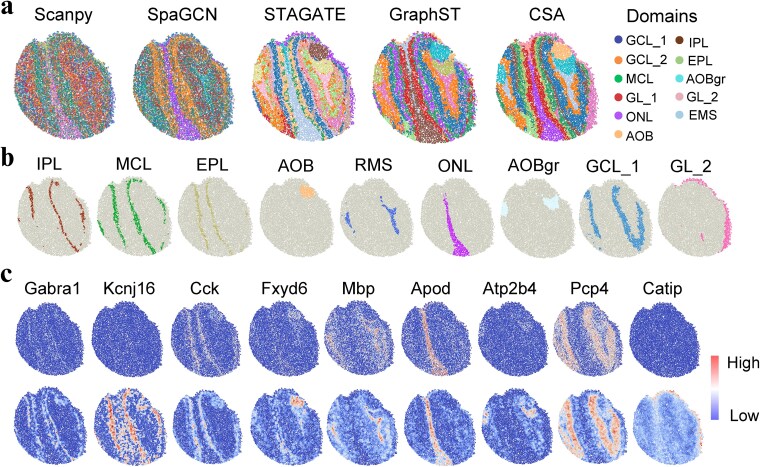
Description of the laminar organization in the mouse olfactory bulb tissue by CSA. (a) Spatial domains identified by Scanpy, SpaGCN, STAGATE, GraphST, and CSA. CSA better identified the narrow laminar organization than other methods. (b) Visualization of the spatial domains identified by CSA. Each identified domain was highlighted in the corresponding color. (c) Comparison of the raw expression levels (top) and the denoised gene expression levels (bottom) of Gabra1, Cck, Fxyd6, Mbp, Apod, Atp2b4, Pcp4, and Catip obtained by CSA. Each marker gene corresponds to the spatial domain in the same column of (b).

We further applied CSA to the spatial transcriptomics of Slide-seqV2 platform to delineate laminar structures of the mouse olfactory bulb (MOB). The laminar structures were annotated by the Allen’s 2D mouse coronal reference atlas [45] including the rostral migratory stream (RMS), granule cell layer (GCL), accessory olfactory bulb (AOB), the granular layer of the accessory olfactory bulb (AOBgr), mitral cell layer (MCL), and olfactory nerve layer (ONL). We applied the Louvain clustering algorithm to embeddings for spatial domain identification. The cluster boundaries revealed by STAGATE were blurry. SpaGCN and Scanpy only approximated the identification of layers such as ONL and GL (Fig. 5a). In contrast, CSA and GraphST detected clear laminar structures than that of other methods. Notably, CSA demonstrated exceptional performance by consistently identifying layers such as GL and GCL_2 and uniquely depicted the elongated and narrow region of the MCL, while GraphST cannot clearly distinguish these structures. GraphCL-E had better performance in detecting spatial domains than GraphCL-F (Supplementary Fig. S9). CSA also exhibited excellent data denoising capabilities for MOB data. Compared to the raw expressions of well-known marker genes for each structure in the MOB, the gene expression profiles denoised by CSA captured distinct spatial patterns, which were consistent with spatial domains (Fig. 5b and c).

## Discussion

In this study, we proposed CSA, which utilizes community-strength-based GraphCL to extract the low dimensional embedding for spatial transcriptomics data. CSA is able to detect spatial domains and denoise the spatial transcriptomics data. We performed several experiments on datasets from different platforms, including 10× Visium, ST, MERFISH, and Slide-seqV2, to evaluate the reliability of CSA. CSA outperform other methods in identifying functional domains.

CSA also takes histology images into consideration. Compared to methods such as SpaGCN, which only integrates histological features by extending one dimension of spatial coordinates, CSA succeeds in depicting a clear histology boundary. The superiority of CSA is mainly attributed to the community-strength-enhanced graph contrastive training strategy.

Though the top-tier performance comparing to the other benchmark methods has been achieved, there is still some space needed to improve the ability of CSA. Firstly, CSA is an unsupervised method, which does not take advantage of the available annotated information. Secondly, more efficient method in extracting features of histology images is still needed in the future. Finally, large memory utilization is a critical issue for spatial transcriptomics data with millions of spots.

**Key Points**

- CSA adopts community-strength-based graph contrastive learning to extract the low dimensional embedding for spatial transcriptomics data.
- Benchmark analyses on the spatial transcriptomic datasets coming from different platforms demonstrate CSA has superior performance in identifying spatially functional domains.
- CSA can adaptively integrate histology image with gene expression profile and effectively denoising the spatial transcriptomics data.

## Supplementary Material

Supplementary_file_bbaf540

## Data Availability

CSA is publicly available as a Python package. The tutorials have been deposited at the link (https://github.com/WHUhuangke/CSA).Author contributions L.Z. conceived the project and supervised the research. L.Z., K.H. and W. T. developed and validated the method. K.H. and L.Z. wrote the manuscript. All authors read and approved the final paper
