# Supplementary material for: Spatial transcriptomic data denoising and domain identification by a community strength-augmented graph autoencoder
Source: Brief Bioinform. 2025 Oct 10;26(5):bbaf540. doi: 10.1093/bib/bbaf540 (PMC12513172; doi:10.1093/bib/bbaf540)
Supplement: Supplementary_file_bbaf540 [file supplementary_file_bbaf540.pdf]

Supplementary for

Spatial transcriptomic data denoising and domain identification by a community strength-augmented graph autoencoder

Ke Huang, Wenqian Tu and Lihua Zhang

Supplementary Text

Below are the pseudocode representations of the CAV algorithm and CED algorithm.

Algorithm1 Communal Attribute Voting (CAV)

Input:

$\mathbf{X}$: Gene expression matrix.

C: number of communities.

$\boldsymbol{I} \in \{0, 1\}^{n \times |C|}$: indicator matrix.

$\boldsymbol{\lambda}_a^{(1)}, \boldsymbol{\lambda}_a^{(2)}$: hyperparameters for Bernoulli distributions.

$\mathbf{S}$: the strength of each community.

Output:

$\widetilde{\mathbf{X}}^{(1)}, \widetilde{\mathbf{X}}^{(2)}$: Corrupted gene expression matrix.

Step 1: Compute the community penalty for each gene.

For each gene a in X:

$$\mathrm{p_a} = \overline{\mathrm{n_a}}(\log(\mathrm{abs}(\mathrm{X})\mathrm{IS}))$$

Step 2: Compute 2 different corruption levels for each gene.

For each gene a in X:

$$\mathrm{m}_a^{(1)} \sim \mathrm{Bernoulli}(1 - \mathrm{p_a}\,\lambda_a^{(1)})$$

$$\mathrm{m}_a^{(2)} \sim \mathrm{Bernoulli}(1 - \mathrm{p_a}\,\lambda_a^{(2)})$$

Step 3: Corrupt the gene expression matrix to obtain 2 corrupted matrix.

$$\widetilde{\mathrm{X}}^{(1)} = \mathrm{m}_a^{(1)} \odot \mathrm{X}$$

$$\widetilde{\mathbf{X}}^{(2)} = \boldsymbol{m}_a^{(2)} \odot \mathbf{X}$$

Algorithm2 Communal Edge Dropping (CED)

Input:

$\mathbf{A}$: Adjacency matrix of the graph.

$\boldsymbol{I}$: Indicator matrix indicating community membership.

$\boldsymbol{\lambda}_{\mathrm{e}}^{(1)}, \boldsymbol{\lambda}_{\mathrm{e}}^{(2)}$: Hyperparameters for Bernoulli distributions.

$\mathbf{S}$**:** the strength of each community.

Output:

$\widetilde{\mathbf{A}}^{(1)}, \widetilde{\mathbf{A}}^{(2)}$: Corrupted adjacency matrix.

Step 1: Compute the edge weight for each edge.

For each edge $e = (v_i, \mathrm{v_j})$ in $\mathbf{A}$:

If $(\boldsymbol{I} \cdot \boldsymbol{I}^{\boldsymbol{T}} \odot \mathbf{A})_{i,j} == 1$

Then $\boldsymbol{p}_e = \widetilde{n_e}(\boldsymbol{I}_i \boldsymbol{S})$

Else $\boldsymbol{p}_e = -\widetilde{n_e}(\boldsymbol{I}_i \boldsymbol{S} + \boldsymbol{I}_j \boldsymbol{S})$

Step 2: Compute the corruption levels for each edge.

For each edge e in $\mathbf{A}$:

$$\mathbf{m}_e^{(1)} \sim Bernoulli(1 - \boldsymbol{p}_{\mathrm{e}} \boldsymbol{\lambda}_{\mathrm{e}}^{(1)}), \mathbf{m}_e^{(2)} \sim Bernoulli(1 - \boldsymbol{p}_{\mathrm{e}} \boldsymbol{\lambda}_{\mathrm{e}}^{(2)})$$

Step 3: Corrupt the adjacency matrix

$$\widetilde{\mathbf{A}}^{(1)} = [(\mathbf{m}_{\mathrm{e}}^{(1)})_{\mathrm{j,k}} \mathbf{A}_{\mathrm{j,k}}^{(1)}],\ \widetilde{\mathbf{A}}^{(2)} = [(\mathbf{m}_{\mathrm{e}}^{(2)})_{\mathrm{j,k}} \mathbf{A}_{\mathrm{j,k}}^{(2)}]$$

Supplementary Figures

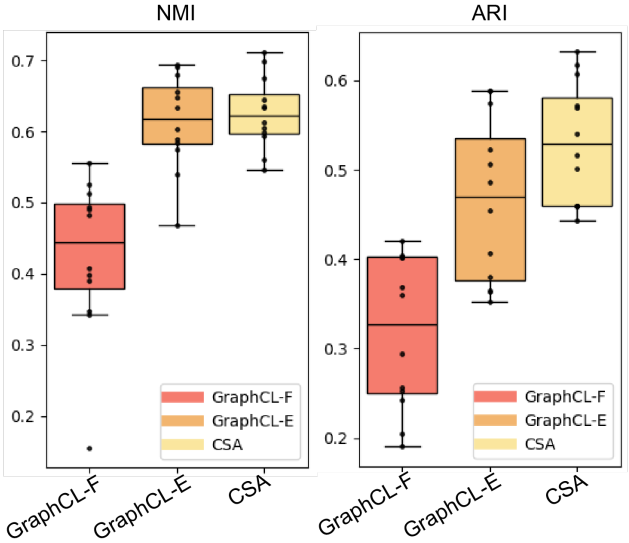

Figure S1. Ablation study of CSA on DLPFC dataset. NMI scores (left) and ARI scores (right) of domain identification by mclust on embeddings of CSA, GraphCL-F and GraphCL-E across 12 slices. GraphCL-F represents replacing node's feature masking method of CSA using randomly node masking strategy in GraphCL. Similarly, GraphCL-E represents replacing edge permuting method of CSA using edge permuting method in GraphCL.

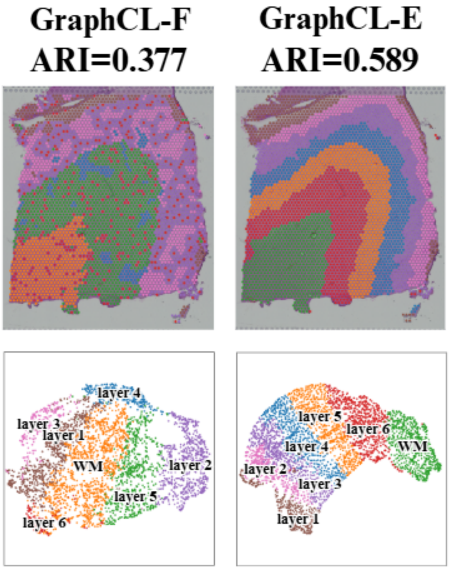

Figure S2. Spatial visualization (top) and UMAP visualization (bottom) of the domain identification results on section 151673 by GraphCL-F and Graph-E.

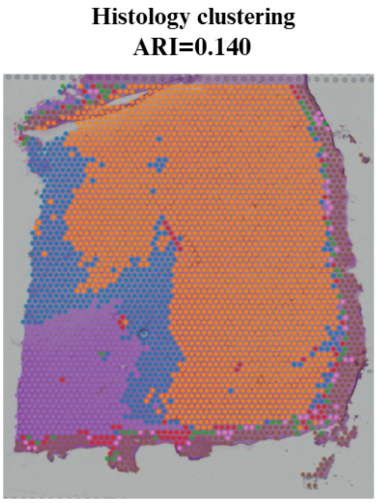

Figure S3. Spatial clustering using raw histology information in slice 151673 of DLPFC dataset.

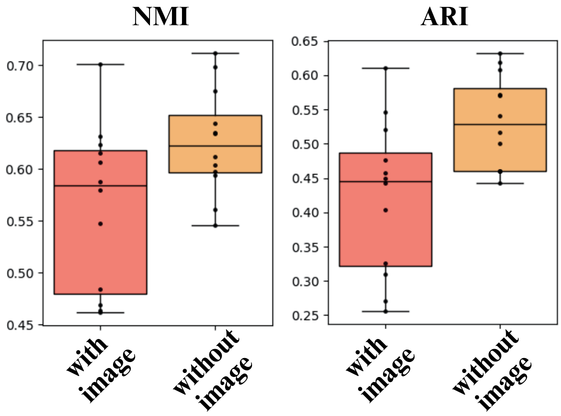

Figure S4. NMI scores (left) and ARI scores (right) of domain identification by mclust on embeddings of CSA with image and CSA without image across 12 slices.

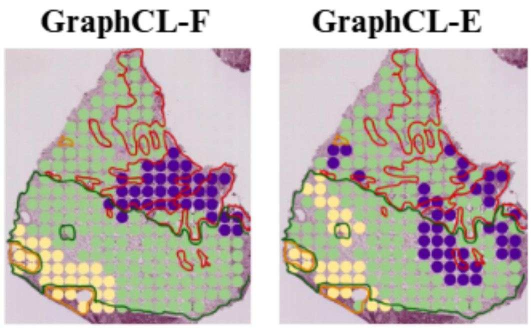

Figure S5. Spatial visualization of the domain identification results on human primary pancreatic cancer tissue data by GraphCL-F and Graph-E.

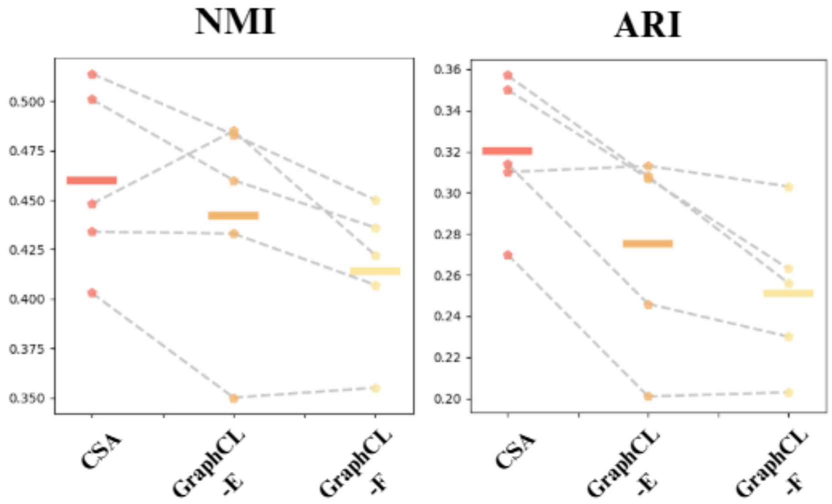

Figure S6. NMI scores (left) and ARI scores (right) of domain identification by mclust on embeddings of CSA, GraphCL-E and GraphCL-F on MERFISH mouse hypothalamic dataset across 5 slices.

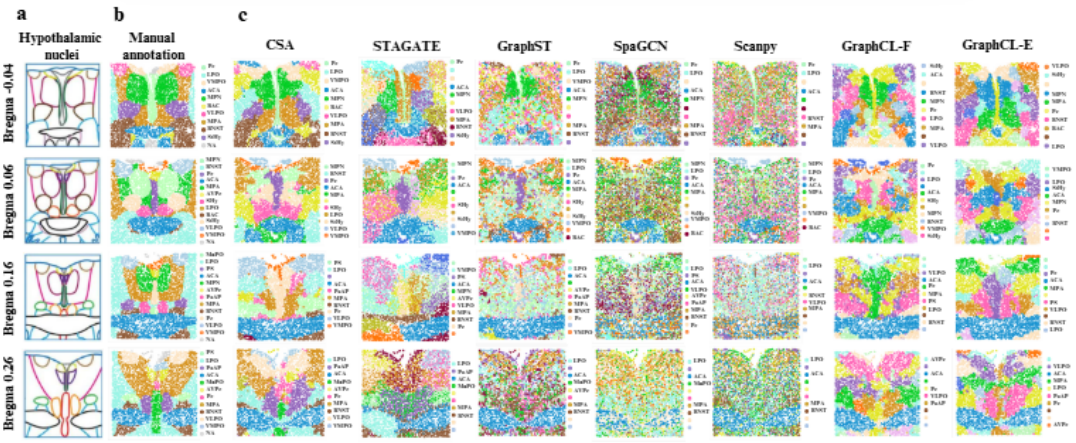

Figure S7. Spatial visualization of the domains identified by CSA, STATAGE, GraphST, SpaGCN, Scanpy, GraphCL-F and GraphCL-E across Bregma -0.04, 0.06, 0,16, 0.26 of mouse hypothalamic dataset.

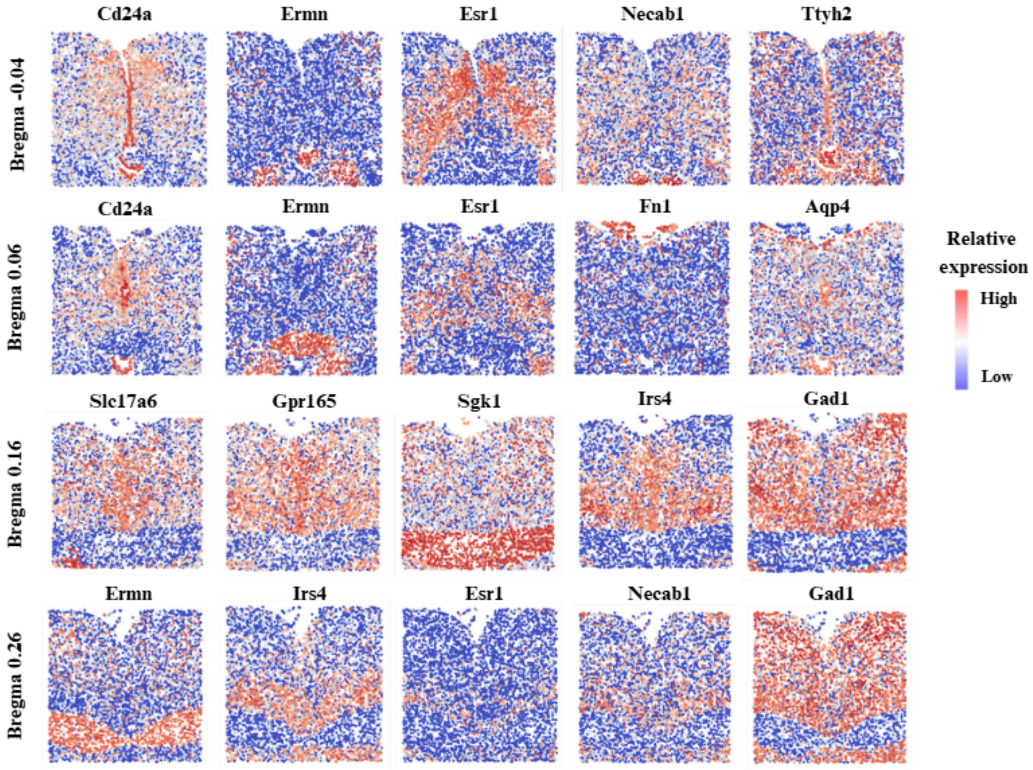

Figure S8. Marker genes for domains in Bregma -0.04, 0.06, 0,16, 0.26 of mouse hypothalamic dataset.

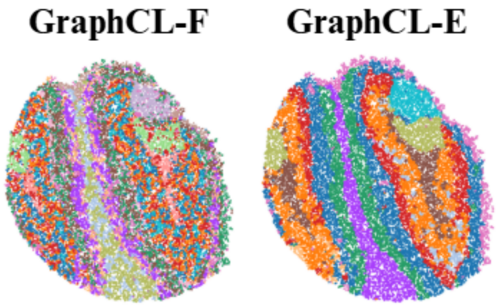

Figure S9. Spatial visualization of the domain identification results on the spatial transcriptomics data of the mouse olfactory bulb tissue by GraphCL-F and Graph-E.

Supplementary Tables

Supplementary Table S1. The running time analyses of CSA on the datasets.

Datasets	Scale	Edges	Time (s/100 epochs)	Memory (MB)
DLPFC (slice 151673)	3639×3000	21,418	2.18	662.82
Mouse olfactory bulb	20139×3000	119,872	42.9	11953.36
Human primary pancreatic cancer	224×3000	1,258	2.9	46.43
Healthy mouse hypothalamic preoptic	5926×161	35,034	2.8	1010.89
